# Persistence of SARS-CoV-2-IgG antibody durability in convalescent COVID-19 patients 6 months after the natural infection

**DOI:** 10.3389/fmed.2025.1623509

**Published:** 2025-08-11

**Authors:** Qiaoli Hua, Peng Zhang, Shengle Qin, Bing Feng, Bin Xiao, Guangjuan Zheng, Taoyu Ye, Danwen Zheng, Jiayi Mo, Yuntao Liu, Yun Cai, Xiaohua Xu, Ji Hu, Banghan Ding, Yingrui Li, Jianwen Guo, Jun Wang, Hongzhi Cao, Zhongde Zhang

**Affiliations:** ^1^Department of Clinical Laboratory, Shenzhen Traditional Chinese Medicine Hospital, The Fourth Clinical Medical College of Guangzhou University of Chinese Medicine, Shenzhen, China; ^2^iCarbonX (Zhuhai) Company Limited, Zhuhai, China; ^3^The Second Clinical College, Guangzhou University of Chinese Medicine, Guangzhou, China; ^4^Department of Pharmacology of Traditional Chinese Medicine, The Second Affiliated Hospital of Guangzhou University of Chinese Medicine, Guangzhou, China; ^5^Department of Emergency, The Second Affiliated Hospital of Guangzhou University of Chinese Medicine, Guangzhou, China; ^6^Guangdong Provincial Key Laboratory of Research on Emergency in TCM, Guangzhou, China; ^7^Shenzhen Digital Life Institute, Shenzhen, China; ^8^Department of Digital Health, South China Hospital of Shenzhen University, Shenzhen, China; ^9^Faculty of Health and Medical Sciences, University of Surrey, Guildford, United Kingdom; ^10^State Key Laboratory of Quality Research in Chinese Medicines, Macau Institute for Applied Research in Medicine and Health, Macau University of Science and Technology, Macau, China; ^11^State Key Laboratory of Dampness Syndrome of Chinese Medicine, The Second Affiliated Hospital of Guangzhou University of Chinese Medicine, Guangzhou, China

**Keywords:** COVID-19, GWAS, SARS-CoV-2, IgG, natural infection

## Abstract

**Introduction:**

Long-term SARS-CoV-2-IgG antibody durability after natural infection remains a critical determinant of long-term protection. However, the factors that affect long-term IgG antibody durability are not fully understood.

**Methods:**

This study delves into the clinical and host genetic factors influencing the level of long-term anti-SARS-CoV-2-receptor-binding domain IgG (RBD-IgG) antibodies after natural infection during the first wave of the COVID-19 pandemic (17 January to 24 June 2020). The cohort, comprising 572 COVID-19 patients from Wuhan, China, had no exposure to COVID-19 vaccines, variants, or antiviral treatments, enabling a focused analysis of the virus’s direct impact.

**Results:**

We found that the rate of RBD-IgG seropositivity 6 months after infection remained high (94.58%). Through a generalized linear model and mediation analysis, older age, independent of disease severity, was found to be a key independent factor associated with higher post-infection RBD-IgG titers. Hypothesis-generating analyses through a genome-wide association study revealed that rs117929853 (*p* = 3.6 × 10^−8^), a variant located upstream of the *xanthine dehydrogenase gene (XDH)*, was significantly associated with RBD-IgG persistence, suggesting a potential mechanistic link between *XDH* polymorphisms and sustained humoral immunity.

**Conclusion:**

The study underscores the significant role of age and genetic factors in the pathogenesis of sustained humoral immunity, which requires further validation.

## 1 Introduction

The durability of humoral immunity following SARS-CoV-2 infection remains a critical determinant of long-term protection. Existing studies have reported conflicting results on the durability of serum antibodies post-SARS-CoV-2 infection in COVID-19 from 4 to 12 months after infection (1–3). The serological trajectory of anti-SARS-CoV-2 IgG antibodies following natural infection demonstrates paradoxical associations with host factors across clinical studies (4, 5). For example, advanced age correlates with elevated baseline antibody titers in some cohorts, potentially due to cumulative immune experience (4), but paradoxically accelerates antibody decay in others (5). These discrepancies may stem from confounding factors, including heterogeneous vaccine coverage, viral evolution patterns, and therapeutic interventions across study populations. Crucially, there remains a paucity of data characterizing antibody maintenance in unvaccinated populations unaffected by subsequent variant exposures—a knowledge gap essential for delineating the genuine immunological imprint of ancestral SARS-CoV-2 infection. Therefore, there is a critical need to understand the immune response after natural infection in convalescent patients.

Utilizing integrated demographic, laboratory test parameters, and genomic data, our study addresses critical knowledge gaps through an integrated analysis of 572 unvaccinated convalescents from Wuhan’s ancestral virus wave (January–June, 2020)—a pivotal period preceding the emergence of SARS-CoV-2 variants, antiviral therapies, and COVID-19 vaccines. This virologically pristine cohort, entirely unexposed to vaccines/variants/therapeutics, enables a definitive assessment of host-intrinsic factors governing 6-month anti-SARS-CoV-2-receptor-binding domain IgG (RBD-IgG) antibody persistence. Combining genome-wide genotyping with deep clinical phenotyping, we pursued two objectives: (1) to conduct correlational analyses between basic phenotypes (age, sex, and disease severity) and RBD-IgG titer and (2) to identify potential genetic loci linked to RBD-IgG through genome-wide association analysis (GWAS).

## 2 Materials and methods

### 2.1 Participants

We recruited 572 recovered COVID-19 patients aged ≥18 years using a dual-phase approach between 28 August and 30 September 2020. In the first phase, 2,228 adult COVID-19 convalescents hospitalized between December 2019 and April 2020 were randomly selected from the electronic medical records of three designated hospitals: Hubei Integrated Chinese and Western Medicine Hospital (*n* = 682), Leishenshan Hospital (*n* = 1,014), and Hankou Hospital (*n* = 532). Recruitment invitations dispatched via SMS yielded 445 consenting participants. Concurrently, media announcements targeting the broader population of COVID-19 survivors supplemented recruitment with an additional 127 volunteers. The final cohort of 572 recovered COVID-19 patients had been discharged from Hubei Provincial Hospital of Traditional Chinese and Western Medicine (*N* = 197), Wuhan’s Leishenshan Hospital (*N* = 165), Wuhan’s Huoshenshan Hospital (*N* = 69), Wuhan’s Hankou Hospital (*N* = 82), and Wuhan’s 15 other hospitals (*N* = 59) between 17 January and 24 June 2020. All patients were interviewed face-to-face between 28 August and 30 September 2020, at the Hubei Provincial Hospital of Traditional Chinese and Western Medicine. The study was approved by the Ethics Committee of Guangdong Provincial Hospital of Chinese Medicine (BF2022-046-01). Written informed consent was obtained from all participants. The study was performed in accordance with the Declaration of Helsinki.

### 2.2 Data sources

**Figure 1:**
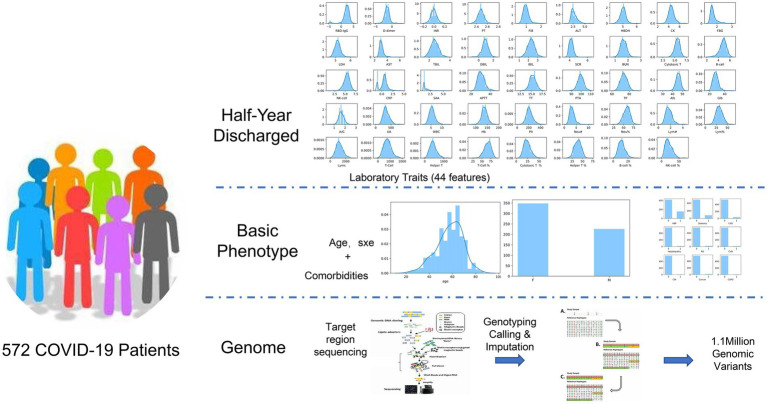
Clinical information and sample of COVID-19 patients for the laboratory traits. INR, international normalized ratio; PT, prothrombin time; FIB, fibrinogen; ALT, alanine aminotransferase; HBDH, α-hydroxybutyric dehydrogenase; CK, creatine kinase; FBG, fasting blood glucose; LDH, lactate dehydrogenase; AST, aspartate aminotransferase; TBIL, total bilirubin; DBIL, direct bilirubin; IBIL, indirect bilirubin; SCR, serum creatinine; BUN, blood urea nitrogen; cytotoxic T, CD3^+^/CD8^+^ lymphocyte count; B-cell, B lymphocyte count; NK-cell, NK lymphocyte count; CRP, C-reactive protein; SAA, serum amyloid A; APTT, activated partial thromboplastin time; TT, thrombin time; PTA, prothrombin activity; TP, total protein; Alb, albumin; Glb, globulin; A/G, albumin/globulin ratio; UA, serum uric acid; WBC, white blood cell count; Hb, hemoglobin; Plt, platelet count; Neu#, neutrophil count; Lym#, lymphocyte count; Lym%, lymphocyte percentage; Lymc, lymphocyte count; for comorbidities, HBP, high blood pressure; CAD, coronary artery disease; RD, respiratory disease; CVD, cardiovascular disease; CKD, chronic kidney disease; and COPD, chronic obstructive pulmonary disease. 0 indicates no, and 1 indicates yes.

We collected data across three broad classes of characteristics (Figure 1 and Table 1): (1) 44 laboratory traits during the recovery period; (2) demographic variables (age and sex), clinical information from the acute phase (disease severity and comorbidities) recorded in hospital medical records, and the duration from discharge to follow-up; and (3) genomic data.

**Table 1:** Clinical characteristics of the enrolled patients.

Categories	All patients (*n* = 572)	Men (*n* = 226)	Women (*n* = 346)	Severe (*n* = 113)	Non-severe (*n* = 459)
Age, years [95%CI]	57.66 [35.24, 80.08]	58.60 [34.51, 82.69]	57.04 [35.83, 78.25]	60.88 [40.11, 81.64]	56.86 [34.30, 79.43]
Duration from discharge to follow-up, days [95%CI]	194.22 [165.03, 223.41]	193.74 [162.33, 209.12]	194.53 [166.85, 208.08]	191.21 [155.13, 227.30]	194.96 [167.88, 222.03]
Comorbidities, *n* (%)					
Hypertension	156 (27.27%)	80 (35.40%)	76 (21.97%)	29 (25.66%)	127 (27.67%)
Diabetes	84 (14.69%)	41 (18.14%)	43 (12.43%)	17 (15.04%)	67 (14.60%)
Coronary heart disease	37 (6.47%)	18 (7.96%)	19 (5.49%)	6 (5.31%)	31 (6.75%)
Liver diseases	12 (2.10%)	5 (2.21%)	7 (2.02%)	5 (4.42%)	7 (1.53%)
Respiratory diseases	18 (3.15%)	9 (3.98%)	9 (2.60%)	7 (6.19%)	11 (2.40%)
Cerebrovascular diseases	13 (2.27%)	6 (2.65%)	7 (2.02%)	2 (1.77%)	11 (2.40%)
Chronic renal diseases	7 (1.22%)	3 (1.33%)	4 (1.16%)	1 (0.88%)	6 (1.31%)
COPD	7 (1.22%)	7 (3.10%)	0 (0.00%)	1 (0.88%)	6 (1.31%)
Tumor	16 (2.80%)	6 (2.65%)	10 (2.89%)	6 (5.31%)	10 (2.18%)
Average incidence	6.80%	8.60%	5.62%	7.28%	6.68%
At least one comorbidity	238 (41.61%)	117 (51.77%)	121 (34.97%)	53 (46.90%)	185 (40.31%)
Median number [95% CI]	0.61 [−1.10, 2.33]	0.77 [−1.05, 2.60]	0.51 [−1.10, 2.11]	0.65 [−1.02, 2.33]	0.60 [−1.13, 2.33]
SARS-CoV-2 IgG antibody					
Sero-positive, n (%)	541 (94.58%)	212 (93.81%)	329 (95.09%)	108 (95.58%)	433 (94.34%)
IgG titer, COI [95% CI]					
All ages (*n* = 572)	14.43 [−18.08, 46.94]	14.09 [−18.92, 47.09]	14.65 [−17.56, 46.87]	15.91 [−13.58, 45.41]	14.06 [−19.13, 47.26]
<30 (*n* = 9)	6.73 [−1.11, 14.56]	10.09 [5.47, 14.71]	5.76 [−1.96, 13.48]	6.21 [−1.51, 13.93]	10.85 [NA, NA]
30~39 (*n* = 39)	8.47 [−6.35, 23.28]	8.72 [−3.37, 20.80]	8.17 [−9.66, 26.01]	7.92 [−6.43, 22.27]	18.57 [8.59, 28.56]
40~49 (*n* = 77)	13.88 [−19.96, 47.72]	13.28 [−9.40, 35.96]	14.31 [−25.86, 54.48]	13.84 [−22.61, 50.29]	14.05 [−4.85, 32.96]
50~59 (*n* = 174)	14.18 [−11.76, 40.12]	12.68 [−12.49, 37.86]	14.83 [−11.44, 41.11]	14.34 [−12.69, 41.37]	13.43 [−7.06, 33.92]
60~69 (*n* = 200)	15.07 [−19.29, 49.43]	14.03 [−17.16, 45.22]	15.73 [−20.58, 52.04]	14.86 [−19.44, 49.16]	15.81 [−19.14, 50.75]
70~79 (*n* = 61)	18.59 [−29.50, 66.67]	21.62 [−37.95, 81.20]	15.45 [−16.73, 47.63]	17.94 [−33.58, 69.47]	20.40 [−17.59, 58.38]
>79 (*n* = 12)	14.96 [−7.46, 37.38]	13.13 [−3.31, 29.57]	20.45 [−17.66, 58.56]	9.17 [−1.74, 20.08]	23.07 [−2.67, 48.81]

COPD, chronic obstructive pulmonary disease; PTSD, post-traumatic stress disorder; disease severity of COVID-19 was defined according to the Chinese management guideline for COVID-19 (version 4.0–7.0). The severe and critical cases were combined as severe; mild and moderate cases were combined as non-severe. Percentages reflect proportions within each column subgroup; the anti-RBD IgG levels are reported as the cutoff index (COI), which is calculated as the ratio of the sample’s chemiluminescence signal to the assay-specific cutoff value (COI, signal/cutoff).

### 2.3 Genomic DNA preparation and bioanalysis

Genomic DNA was extracted from the peripheral blood of all patients via a standard procedure. Libraries were constructed using a target region enrichment kit that covered approximately 10 M of the genomic region and was sequenced on the Illumina HiSeq X Ten platform.

DNA reads were preprocessed using fastp (6) software and aligned to the GRCh38 reference genome via the BWA-MEM approach. Variant detection was subjected to the GATK (7) best practice workflow (8). The parameter for the VariantFiltration algorithm was set with the filter expression: “QD < 2.0 || MQ < 40.0 || FS > 60.0 || MQRankSum<−12.5 || ReadPosRankSum<−8.0.”

Nine samples with high sequencing depth in this study were selected for saturation analysis to determine the optimal sequencing depth threshold. The cutoff was set at 30x, corresponding approximately to the elbow point of all curves (Supplementary Figure S4).

Population stratification refers to systematic differences in allele frequencies between subpopulations and is a known source of false-positive results in GWAS (9). To address this, the SNP data for 2,504 common individuals from five major human populations were downloaded from the 1,000 Human Genomes Project. SNPs located outside the target region of the enrichment kit or within the MHC region were excluded from the analysis. Principal component analysis (PCA) was performed on the remaining SNP data, and an ethnographic classification model was constructed using the first six PCs and the random forest algorithm. SNPs from all study samples were then passed through this model to identify the human population. Identity-by-descent (IBD) analysis was performed using PLINK. Blood descent-related pairs were defined as those with a Z0 score of < 0.95, and one individual from each pair was excluded from subsequent analyses.

To increase the power of our study, we performed SNP imputation using the ChinaMAP reference panel, which includes more than 10,000 samples belonging to multiple ethnic Chinese subgroups (10). We excluded SNPs with a missing calling ratio of > 5% or loci in the MHC region for better imputation accuracy. Finally, SNPs on the 22 autosomal chromosomes were successfully imputed. After strict quality control, SNPs with an R^2^ score of <0.7, a minor allele frequency of <0.01, or those located in the MHC regions were excluded.

### 2.4 Antibody detection (measurement of RBD-specific IgG antibodies)

Plasma samples were inactivated at 56°C for 30 min before testing. IgG antibodies against the SARS-CoV-2 RBD spike protein were tested with two-step indirect immunoassay electrochemiluminescence immunoassay kits (Antu Biotech Co., Ltd. Henan, China), according to the manufacturer’s instructions (11).

### 2.5 Statistical analysis

Continuous variables were expressed as the mean [mean−1.96*standard deviation, mean+1.96*standard deviation]. Categorical variables were summarized as counts and percentages within each category. Numerical variables were assessed for normality using the Kolmogorov–Smirnov test and skewness calculations. Features with *p*-values of <0.01 and skewness coefficients of >0 were log-transformed for subsequent statistical tests.

Pearson’s correlation analysis was performed between numerical variables, including age, IgG level, and laboratory traits. The Mann–Whitney *U*-test was used to compare the numerical and categorical variables (e.g., IgG level vs. sex, age vs. disease severity). The chi-squared test was used to assess associations between categorical variables.

### 2.6 Correlations among IgG vs. age and disease severity (denoted as severity)

The generalized linear mixed regression analysis was performed using the formula glm [IgG ~ age*severity, family = binomial]. For the mediation analysis, IgG levels (log-transformed) were set as the dependent variable, disease severity as the mediating variable, and age as the independent variable. The mediation analysis was conducted using the “mediate” function of the mediation R package, following the method described in a previous study (12). The *p*-value for the mediation effect was calculated as the average mediation effect obtained from bootstrap testing.

### 2.7 Association of clinical features with human genomic variants

PCA was conducted based on clinical features, including age, sex, severity, nine comorbidities, and the time from hospital discharge to follow-up for all patients. PC1 explained 81.3% of the variance in the entire phenotype matrix. Associations between SNPs and virus-specific IgG titers were analyzed using a simple linear regression model conditioned on PC1. Statistical analyses were performed using the R package (v.3.32.1).

## 3 Results

### 3.1 Sample and data description

Between 17 January and 24 June 2020, 572 convalescents in China who had recovered from COVID-19 caused by wild-type SARS-CoV-2 were assessed 6 months after infection. Data from convalescents, including age, sex, 44 laboratory parameters, and genome target region capture sequencing, were collected during their recovery phase. Additionally, disease severity during the acute phase (from symptom onset to hospital discharge) was extracted from medical records (Figure 1). The subgroup distributions for all clinical traits are shown in Supplementary Figure S1 and Supplementary Figure S2, stratified by sex and COVID-19 severity.

The clinical characteristics of the participants are presented in Table 1. The mean age was 57.66 (95% CI: 35.23 ~ 80.08) years, with 346 (60.49%) women and 226 (39.51%) men. The average duration from discharge to follow-up was 194.22 (95% CI: 165.03–223.41) days. According to the Diagnosis and Treatment Guidelines in China (13), the severity of COVID-19 was classified as mild (*N* = 25, 4.37%), moderate (*N* = 434, 75.87%), severe (*N* = 105, 18.36%), or critical (*N* = 8, 1.40%). To facilitate the analysis of the relationship between disease severity and other features, mild and moderate cases were combined into the non-severe group (*N* = 459, 80.42%), and severe and critical cases were combined into the severe group (*N* = 113, 19.76%). Hypertension was the most common comorbidity (27.27%), followed by diabetes (14.69%). A total of 238 (41.61%) COVID-19 patients suffered from at least one comorbidity.

The ability of the immune system to provide sustained protection to the body after recovery from COVID-19 is a key concern. Consistent with recent studies (14, 15), 94.58% of patients were SARS-CoV-2 IgG seropositive at half a year after discharge (Table 1). Supplementary Figure S3 illustrates the distribution of IgG antibody levels across different age groups. The IgG levels exhibited a consistent upward trend with increasing age between 30 and 79 years.

Genomic data were obtained from these patients using a target region capture sequencing strategy. The data were processed using a frequently used pipeline. After genotyping and imputation with the ChinaMap database as a reference and strict quality control measures (10), 1.10 million high-quality SNPs were obtained for subsequent analyses. Of the 572 recruited patients, 15 individuals who showed a close genetic relationship with others based on the identify-by-descent (IBD) analysis were excluded from subsequent analyses, resulting in a total of 557 unrelated individuals for subsequent analyses.

### 3.2 The basic phenotype associated with RBD-IgG levels 6 months after infection

**Figure 2:**
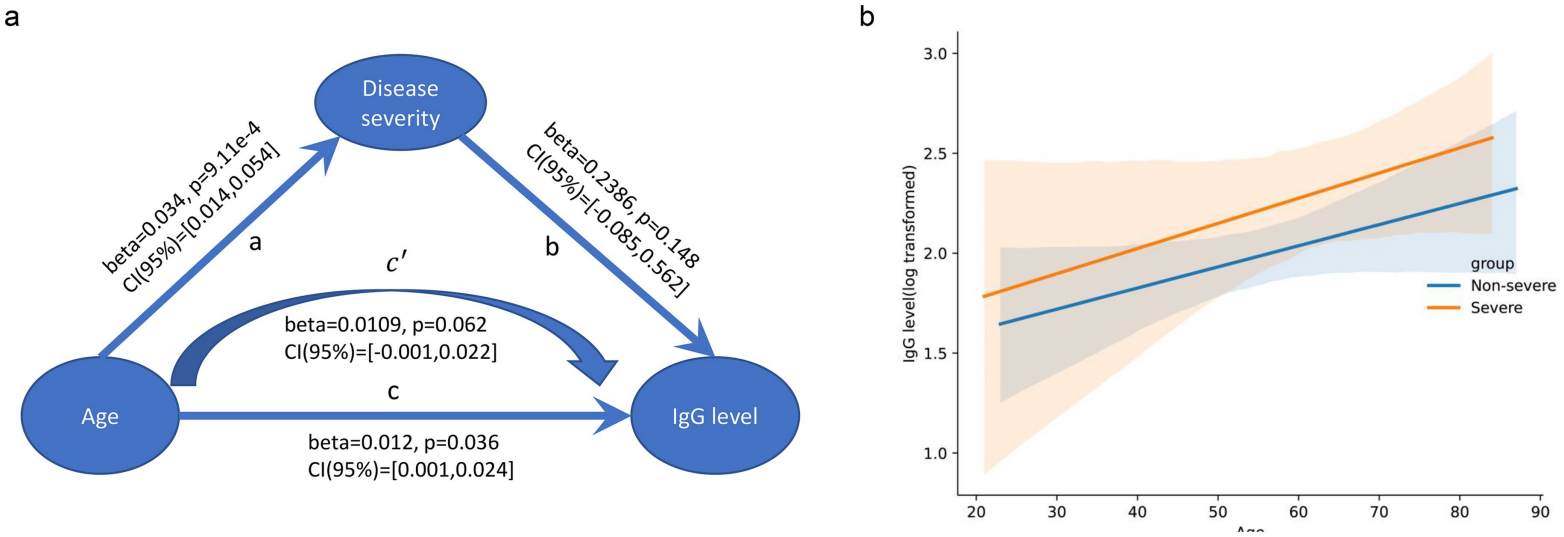
Association between age, disease severity, and RBD-IgG levels. **(a)** Mediation analysis of IgG level vs. age and disease severity. The arrows indicate the direction of the relationships, with coefficients (beta), *p*-values, and 95% confidence intervals (CI) provided for each path. No mediation effect was observed. **(b)** Scatter plot with regression lines shows the relationship between age and log-transformed RBD-IgG levels for the non-severe (blue line) and severe (orange line) groups. The X-axis represents age (years), and the Y-axis represents log-transformed RBD-IgG levels. The shaded areas represent the 95% confidence intervals.

First, we performed an association analysis between the basic phenotypes (age, sex, and disease severity) and RBD-IgG levels. Disease severity (*p* = 0.012) and age (*p* = 0.036) were significantly associated with RBD-IgG levels 6 months after discharge. As age is also associated with disease severity, we conducted a generalized linear model and mediation analysis to investigate the mechanisms underlying these three features. Age (*p* = 0.036), but not disease severity (*p* = 0.148) or the interaction effect of age and disease severity (*p* = 0.895), was a significant independent contributor to the RBD-IgG titer. No mediating effects (*p* = 1) were detected in our data (Supplementary Table S1). Therefore, these analyses proved that older age was an independent contributor to and correlated positively with RBD-IgG levels after recovery, whereas disease severity was not (Figure 2).

We further examined the associations between the 44 laboratory traits and RBD-IgG titers. To our surprise, none of the other laboratory tests showed significant associations (FDR-adjusted *p* < 0.05, Supplementary Table S2).

### 3.3 Genome-wide association analysis of RBD-IgG levels at 6 months after infection

To identify genetic factors associated with RBD-IgG titers, we conducted a GWAS on PC1, explaining 81.3% of the variance in clinical features (Supplementary Figure S4), using a multiple-factor linear model. The inflation factor (*λ*) for RBD-IgG was 1.005 (Supplementary Figure S5), indicating no significant systematic bias. To mitigate false-positive associations due to sporadic genotyping errors, singleton SNPs (including adjacent SNPs imputed from the same variant) were excluded from the analysis. A standard GWAS significance cutoff of 5 × 10^−8^ was applied.

**Figure 3:**
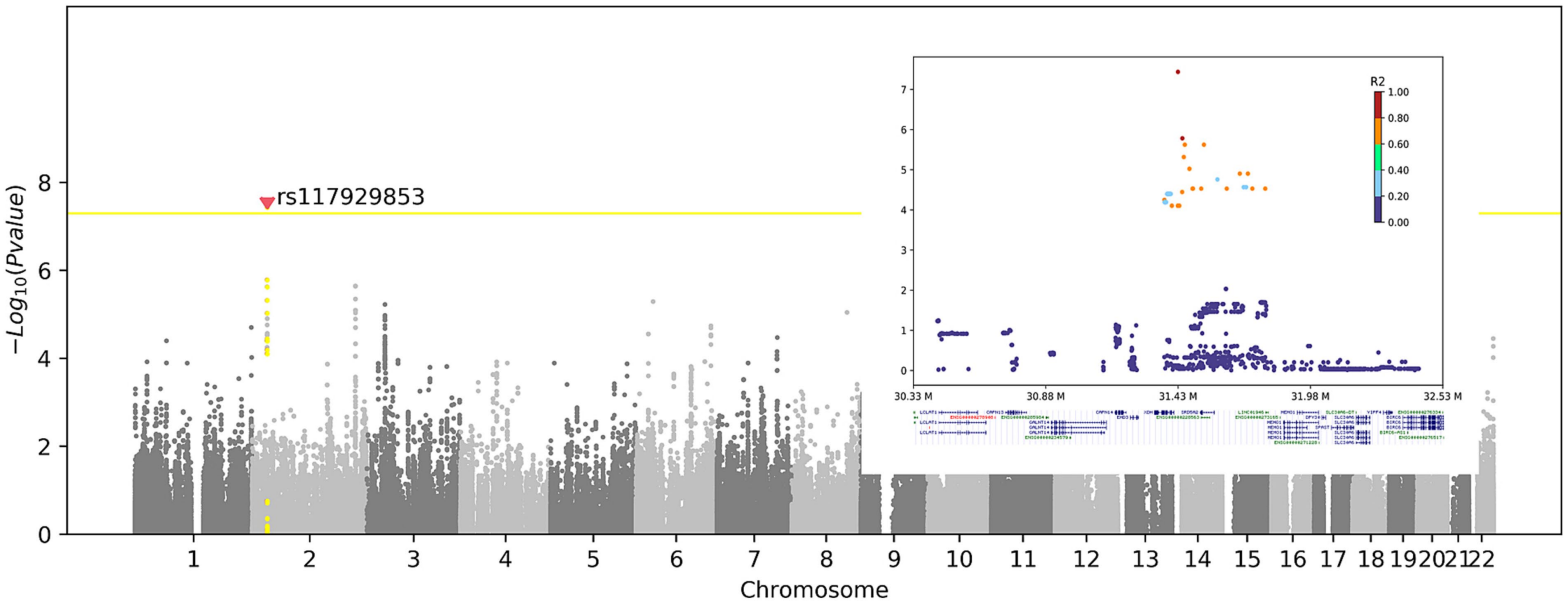
Genome-wide association of the RBD-IgG antibody titers. Left panel: Manhattan plot shows the association between genetic variants and RBD-IgG titers. X-axis: chromosome number; Y-axis: −Log10(*p*-value). Yellow line: genome-wide significance threshold (*p* = 5 × 10^−8^). Red dot: significant SNP rs117929853 (*p* = 3.65 × 10^−8^), upstream of the *XDH* gene. Right panel: Regional association plot of rs117929853. X-axis: genomic position (M); Y-axis: −Log10(*p*-value). Colors: linkage disequilibrium (R^2^) with rs117929853. The *XDH* gene is highlighted below.

First, we checked whether genetic variants could contribute to the RBD-IgG antibody titer. Although the sample size was small, we detected an intergenic SNP, rs117929853 (*p* = 3.65 × 10^−8^), located upstream of the *XDH* gene, which exceeded the significance cutoff (Figure 3). The protein encoded by *XDH* is a critical enzyme involved in the oxidative metabolism of purines, pterin, and aldehydes and is a central component of the innate immune system (16–19). The assessment of IgG antibody levels in individuals with reference and alternate genotypes is shown in Supplementary Figure S6.

## 4 Discussion

This study investigated clinical and host genetic factors influencing long-term IgG antibody durability in 572 COVID-19 convalescents from Wuhan, who were infected at the pandemic onset and unexposed to vaccines, variants, or antiviral agents. This enabled a definitive assessment of the clinical and host factors associated with 6-month IgG persistence. We found that older age, but not disease severity, was an independent factor that was positively correlated with higher SARS-CoV-2 anti-RBD-IgG titers 6 months after natural infection. For the first time, we identified a novel variant (rs117929853) located upstream of *XDH*, a gene that plays an important role in response to oxidative stress and the innate immune system, which was significantly associated with RBD-IgG titer by genome-wide association study.

We found that the rate of RBD-IgG seropositivity remained high (94.58%) 6 months after SARS-CoV-2 natural infection. This finding was consistent with a previous study that revealed that IgG levels remained stable and were detected in 94% of healthcare workers 6 months after infection (20), and the neutralizing antibody activities and memory T cells against SARS-CoV-2 can remain stable for up to 6–7 months post-infection (15). In addition, consistent with previous antibody studies (20–22), older age was associated with higher RBD-IgG titers after recovery. It is possible to hypothesize that this could result from boosting of cross-reactive anti-nucleocapsid antibodies due to prior exposure, for example, to a previously circulating or geographically restricted human coronavirus.

Through GWAS analysis, we report for the first time a novel genomic variant, rs117929853, significantly associated with RBD-IgG titers. This variant is located upstream of the fine-mapped cis-eQTL region (based on GTEx project data) of the *XDH* gene, indicating that it might affect *XDH* expression. Motif analysis indicated that this region overlaps with the predicted binding sites for transcription factors involved in oxidative stress responses. Further functional annotation of rs117929853 revealed that this SNP resides within a region of open chromatin, as evidenced by DNase-I hypersensitive sites in multiple immune-related cell types. This suggests that rs117929853 might influence *XDH* expression through regulatory element modulation. Notably, this region may harbor binding sites for oxidative stress regulators (e.g., NRF2) and immune mediators (e.g., STAT factors) (17). Furthermore, XDH encodes an enzyme involved in purine metabolism that has been linked to innate immune functions (23). Although purine metabolic imbalance can theoretically promote reactive oxygen species (ROS) accumulation (16), oxidative stress has been associated with SARS-CoV-2 infection (19). These observations collectively propose—but do not establish—a speculative mechanistic framework in which rs117929853 might contribute to IgG persistence via oxidative stress pathways, which requires rigorous validation.

While our study focused on the dynamics of IgG antibody levels at 6 months post-infection in a vaccine-naïve cohort, we acknowledge that residual confounding factors, particularly pre-existing immunity to seasonal coronaviruses (sCoVs) and baseline health status beyond documented comorbidities, warrant careful consideration. Pre-existing humoral immunity to sCoVs is highly prevalent in the general population, with seroprevalence rates exceeding 96% for at least one sCoV subtype, notably HCoV-229E and HCoV-OC43 (24). Such immunity could theoretically influence SARS-CoV-2 antibody responses through mechanisms such as “original antigenic sin” (OAS), where pre-existing sCoV-reactive B-cell clones dominate the early immune response (25). In addition, baseline health status, encompassing factors such as body mass index (BMI) and undetected subclinical conditions, may also represent a potential source of residual confounding.

Our study has several limitations. First, while the identified genetic association between rs117929853 and anti-RBD IgG persistence has reached genome-wide significance, the modest sample size for GWAS necessitates a cautious interpretation of its role in humoral immunity. The GWAS findings require validation in larger cohorts to confirm effect size stability and exclude potential confounding factors by population stratification. Second, the proposed mechanistic link between XDH and IgG persistence remains speculative. Our study lacks direct experimental validation of this mechanistic link, which renders this link speculative. We explicitly present this mechanism as a testable hypothesis requiring future functional validation. Third, no independent validation cohort was used in our study. The absence of an independent validation cohort restricts the generalizability of our findings and precludes confirmation of the variant’s effects across diverse populations. Fourth, participants were enrolled via a voluntary participation mechanism, which may introduce potential selection bias, as self-selected participants likely represent a health-conscious subgroup with greater healthcare engagement and concern for long-term outcomes, potentially limiting generalizability to less health-engaged populations. Fifth, the cohort was exclusively derived from a Chinese population, further limiting the extrapolation to other ethnic groups. Sixth, total IgG levels were not quantified, hindering a comprehensive assessment of systemic humoral immunity despite our focus on SARS-CoV-2-specific anti-RBD IgG responses. Further studies are needed to enhance statistical power through larger sample sizes, validate the findings in independent multi-ethnic cohorts, and incorporate total IgG measurements to comprehensively address the broader immunological implications of the identified genetic associations.

## 5 Conclusion

Older age and the genomic variant rs117929853 were significantly associated with RBD-IgG titers 6 months post-infection. The mechanistic link between rs117929853, located near the *XDH* gene, and IgG persistence remains speculative and requires further validation.

## Data Availability

The datasets that support the findings of this study have been deposited in Human Genetic Resources Information Management Platform administered by the Ministry of Science and Technology of China (*BF2022051211129).
