# Supplementary material for: Persistence of SARS-CoV-2-IgG antibody durability in convalescent COVID-19 patients 6 months after the natural infection
Source: Front Med (Lausanne). 2025 Aug 11;12:1623509. doi: 10.3389/fmed.2025.1623509 (PMC12375572; doi:10.3389/fmed.2025.1623509)
Supplement: Supplementary file 1 [file Data_Sheet_1.zip › Supplementary file/Supplementary Figures-revised.docx]

**Supplement for “Persistence of SARS-CoV-2-IgG Antibody Durability in convalescent COVID-19 patients 6 months after the natural infection”**

**
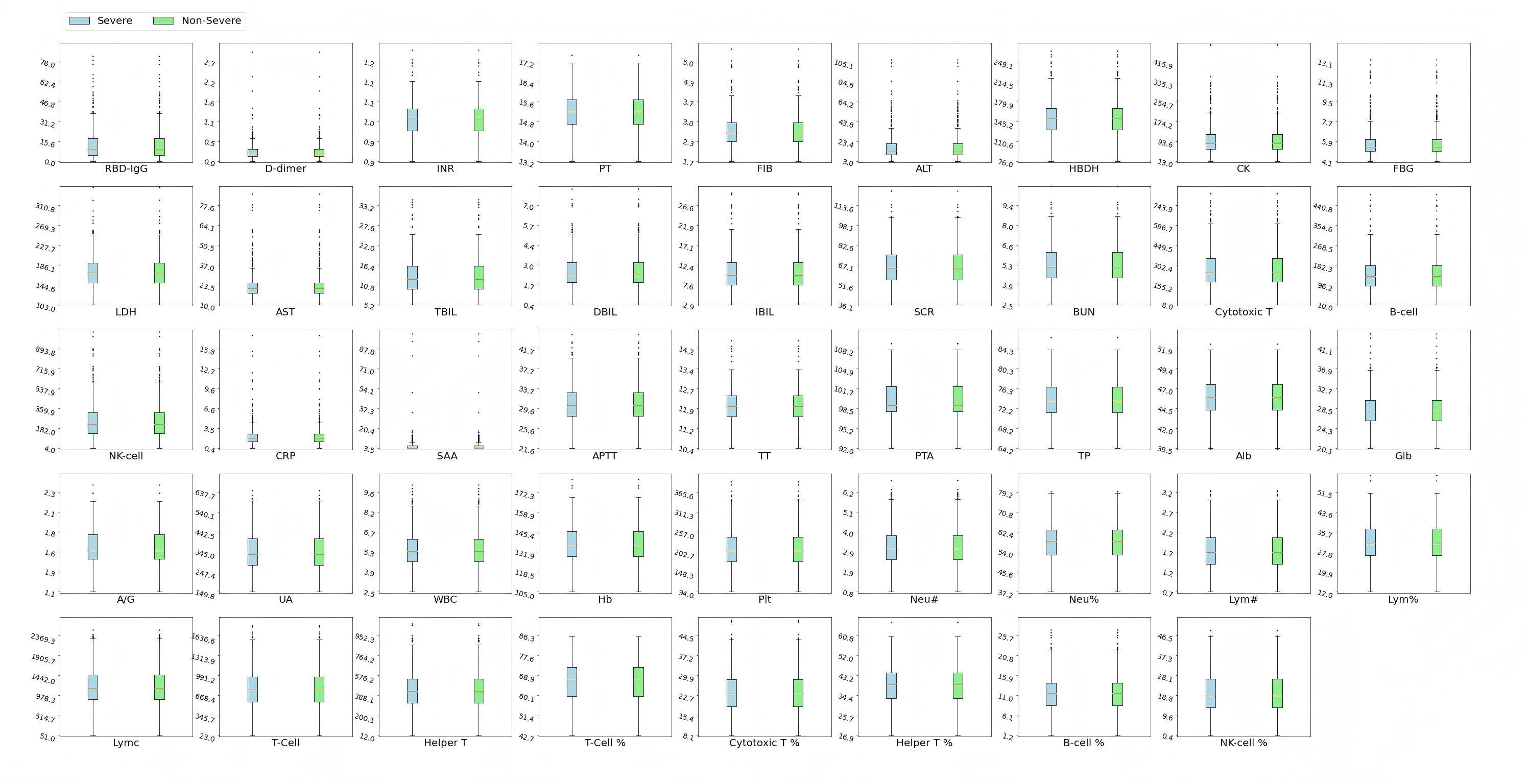
**

**Figure S.1. Laboratory traits of enrolled patients 6 months after natural infection grouped by disease severity**

For the laboratory traits, INR = International normalized ratio; PT = Prothrombin time; FIB = Fibrinogen; ALT = Alanine aminotransferase; HBDH = α-Hydroxybutyric dehydrogenase; CK = Creatine kinase; FBG = Fasting blood glucose; LDH = Lactate dehydrogenase; AST = Aspartate aminotransferase; TBIL = Total bilirubin; DBIL = Direct bilirubin; IBIL = Indirect bilirubin; SCR = Serum creatinine; BUN = Blood urea nitrogen; Cytotoxic T = CD3+/CD8+ lymphocyte count; B-cell = B lymphocyte count; NK-cell = NK lymphocyte count; CRP = C-reactive protein; SAA = Serum amyloid A; APTT = Activated partial thromboplastin time; TT = Thrombin time; PTA = Prothrombin activity; TP = Total protein; Alb = Albumin; Glb = Globulin; A/G = Albumin/globulin ratio; UA = Serum uric acid; WBC = White blood cell count; Hb = Hemoglobin; Plt = Platelet count; Neu# = Neutrophil count; Neu% = Neutrophil percentage; Lym# = Lymphocyte count; Lym% = Lymphocyte percentage; Lymc = Lymphocyte count;

**
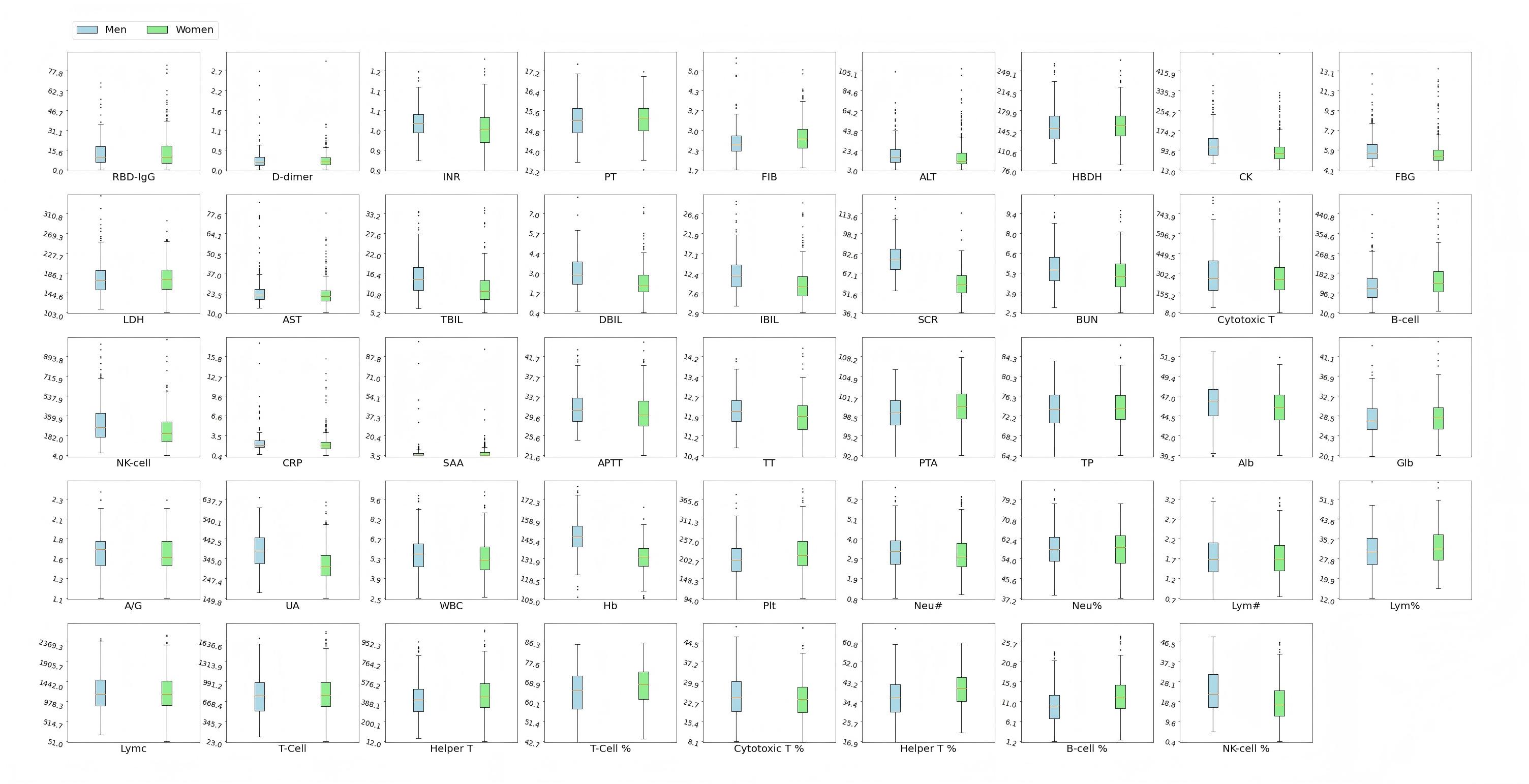
**

**Figure S.2. Laboratory traits of enrolled patients 6 months after natural infection grouped by sex**

For the laboratory traits, INR = International normalized ratio; PT = Prothrombin time; FIB = Fibrinogen; ALT = Alanine aminotransferase; HBDH = α-Hydroxybutyric dehydrogenase; CK = Creatine kinase; FBG = Fasting blood glucose; LDH = Lactate dehydrogenase; AST = Aspartate aminotransferase; TBIL = Total bilirubin; DBIL = Direct bilirubin; IBIL = Indirect bilirubin; SCR = Serum creatinine; BUN = Blood urea nitrogen; Cytotoxic T = CD3+/CD8+ lymphocyte count; B-cell = B lymphocyte count; NK-cell = NK lymphocyte count; CRP = C-reactive protein; SAA = Serum amyloid A; APTT = Activated partial thromboplastin time; TT = Thrombin time; PTA = Prothrombin activity; TP = Total protein; Alb = Albumin; Glb = Globulin; A/G = Albumin/globulin ratio; UA = Serum uric acid; WBC = White blood cell count; Hb = Hemoglobin; Plt = Platelet count; Neu# = Neutrophil count; Neu% = Neutrophil percentage; Lym# = Lymphocyte count; Lym% = Lymphocyte percentage; Lymc = Lymphocyte count;


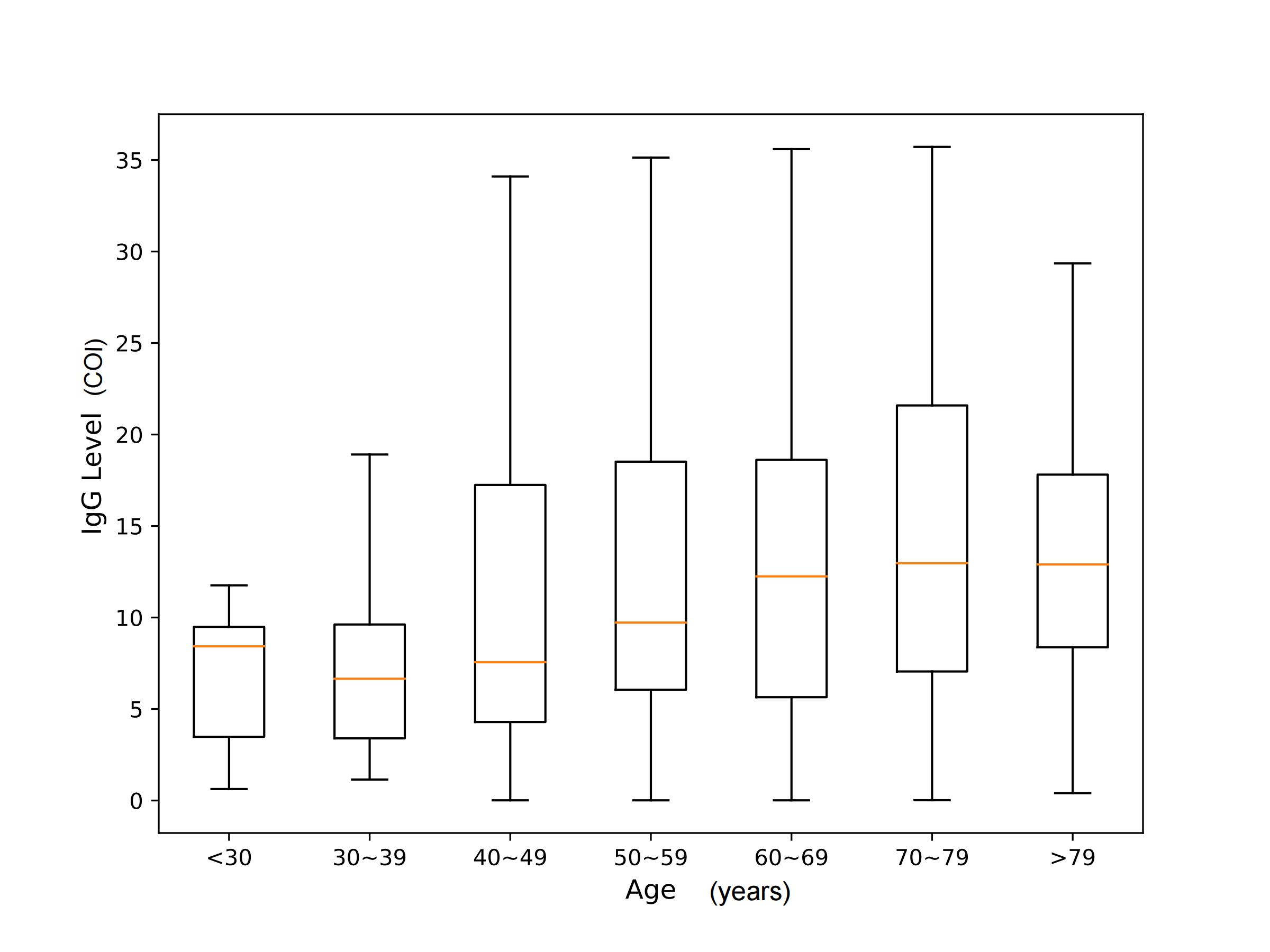


**Figure S.3 The distribution of IgG levels across different age groups.**


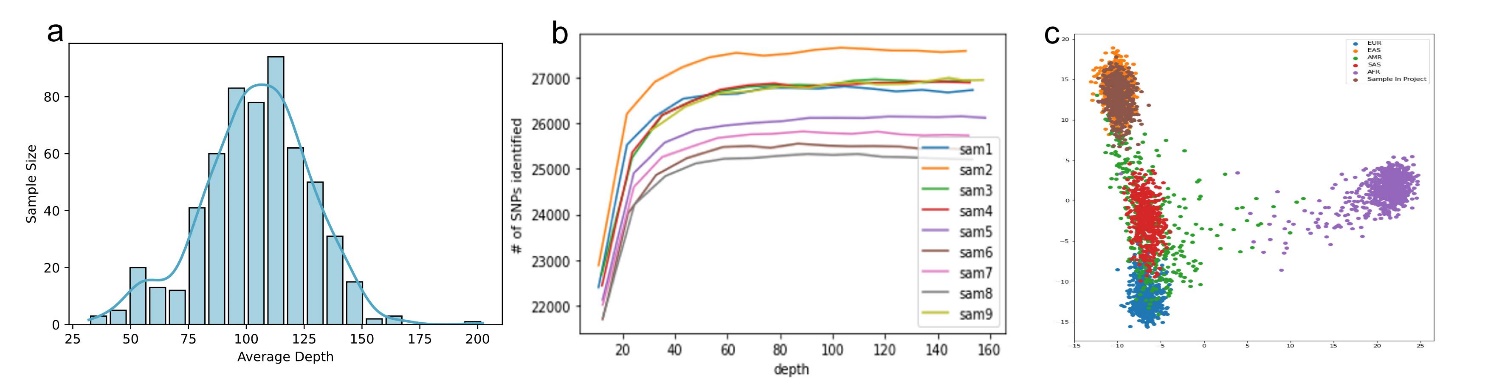


**Figure S4. Plots for genome-wide association analysis**

**a** Distribution of average depth for all samples in this project; **b** Saturation analysis for 9 samples with high sequencing depth; **c** Principal component analysis for samples from different populations;


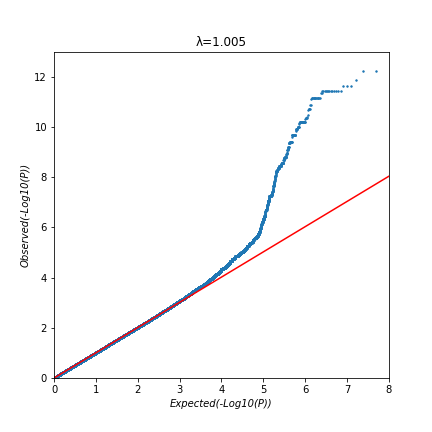


**Figure S.5. The inflation factor (λ) for RBD-IgG**


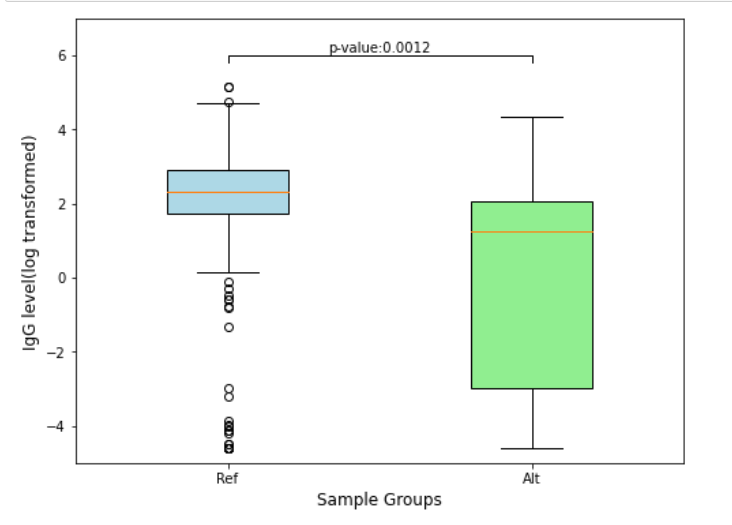


**Figure S.6. Assessment of IgG antibody levels in individuals with reference and alternate genotypes**

Ref：Reference group (homozygous carriers of the reference allele (wild-type)

Alt: Alternate group (carriers of the alternate allele)

The ****Ref** group** present with higher anti-RBD IgG levels compared with ****Alt** group** (***P* = 0.0012**).
